# Charge Transport in Water–NaCl Electrolytes with Molecular Dynamics Simulations

**DOI:** 10.1021/acs.jpcb.2c08047

**Published:** 2023-03-15

**Authors:** Øystein Gullbrekken, Ingeborg Treu Røe, Sverre Magnus Selbach, Sondre Kvalvåg Schnell

**Affiliations:** † Department of Materials Science and Engineering, Norwegian University of Science and Technology, NTNU, Trondheim NO-7491, Norway

## Abstract

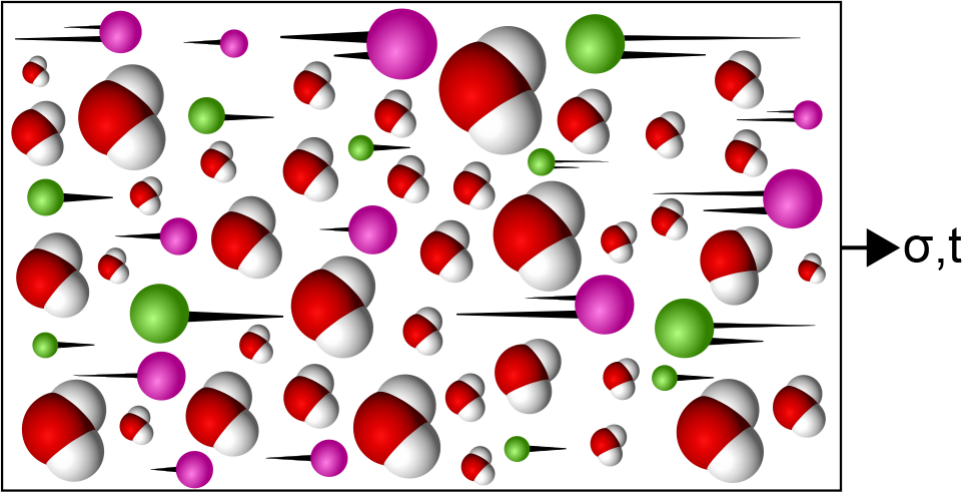

A systematic description of microscopic mechanisms is necessary to understand mass transport in solid and liquid electrolytes. From Molecular Dynamics (MD) simulations, transport properties can be computed and provide a detailed view of the molecular and ionic motions. In this work, ionic conductivity and transport numbers in electrolyte systems are computed from equilibrium and nonequilibrium MD simulations. Results from the two methods are compared with experimental results, and we discuss the significance of the frame of reference when determining and comparing transport numbers. Two ways of computing ionic conductivity from equilibrium simulations are presented: the Nernst–Einstein approximation or the Onsager coefficients. The Onsager coefficients take ionic correlations into account and are found to be more suitable for concentrated electrolytes. Main features and differences between equilibrium and nonequilibrium simulations are discussed, and some potential anomalies and critical pitfalls of using nonequilibrium molecular dynamics to determine transport properties are highlighted.

## Introduction

Electrolytes have a central place in many disciplines, including electrochemistry, biology, and biomedical applications.^1^ Technologies for energy production and storage are required to solve current challenges with global warming and the transition to sustainable energy sources.^2^ The increased use of renewable and sustainable, but intermittent, energy sources depends on temporary storage of energy in batteries or hydrogen for fuel cells. Next-generation rechargeable batteries with high energy and power density require new electrode materials and new electrolytes with improved transport properties and improved electrochemical stability.^3−5^

Ionic conductivity and transport numbers are important with respect to charge transport in electrolytes. The ionic conductivity relates the ability of the electrolyte to carry electric charge through ionic motion,^1^ while the ion transport number is defined as the fraction of the total current carried by the ionic species in question. Both properties are defined in the absence of concentration gradients.^1^ For energy storage applications, e.g., a Li-ion battery electrolyte, the Li-ion transport number should preferably be as high as possible, ideally close to unity to improve the rate performance.^6^

Equilibrium or nonequilibrium molecular dynamics (MD) simulations can be used to compute ionic conductivity and transport numbers in complex mixtures. In nonequilibrium simulations, a flux of particles, energy, or charge is established by creating a gradient in the simulation box, or alternatively by applying an external field.^7^ Ionic conductivity can be calculated directly from the particle displacements as a function of the gradient or field strength. From equilibrium simulations in the canonical or microcanonical ensemble, transport properties can be obtained by sampling the particle displacements, current density, or velocities, using the Einstein^8^ or Green–Kubo^9^ relations.

Several experimental methods exist for determining ionic conductivity and transport numbers of electrolytes. Typically, ionic conductivity is measured by electrochemical impedance spectroscopy. Transport numbers in liquid electrolytes can be measured by the Hittorf,^10^ moving boundary,^11^ emf,^12^ or other methods. Transport numbers are always determined with respect to a reference frame. Different methods can employ different reference frames which requires caution when comparing values from different sources.

In this work, we have investigated and compared equilibrium and nonequilibrium MD simulation methods to compute ionic conductivity and transport numbers in model electrolytes. We have examined a benchmark electrolyte mixture which has been extensively studied experimentally: water with different concentrations of solvated NaCl. We have chosen the SPC/E^13^ water model due to its computational efficiency and a force field for solvated NaCl parametrized to reflect the microstructure of sodium chloride solutions.^14^ Additionally, we have studied a polarizable water model, SWM4-NDP,^15^ with polarizable NaCl.^16^ A comparison is made to experimental data. We present potential challenges with using nonequilibrium MD simulations to obtain these quantities and discuss the implications.

## Theory

Charge transport properties can be obtained directly from equilibrium molecular dynamics simulations by sampling the particle displacements, i.e., the mean-squared displacement (MSD). The general equation for a transport coefficient *D* in three dimensions can be written:^17^
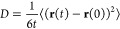
1where *t* is time, **r** is the particle position vector, and ⟨···⟩ denotes the ensemble average. For a multicomponent system, this can be generalized to the self-diffusion coefficient of a component *i*:^8^
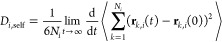
2where *N*_*i*_ is the number of particles of type *i*. The self-diffusion coefficient describes the motion of a single molecule of a specific type, describing the stochastic movements of individual particles, i.e., the movement of particles in the absence of a chemical potential gradient.

One way of computing ionic conductivity and transport numbers in electrolytes from equilibrium MD simulations is by employing the Nernst–Einstein (NE) approximation. The ionic conductivity is then related to the self-diffusion coefficients,^18−23^ i.e., eq 2. The Nernst–Einstein approximations of the ionic conductivity and transport numbers are derived by substituting the expression for ionic mobility in the Nernst–Einstein equation into the equation for ionic conductivity. The Nernst–Einstein equation relates the ionic mobility to the diffusion coefficient:^24^
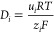
3where *D*_*i*_ is the diffusion coefficient of species *i*, *u*_*i*_ is the mobility of species *i*, *R* is the gas constant, *T* is the temperature, *z*_*i*_ is the charge valency of species *i*, and *F* is Faraday’s constant. The derivation of the NE approximation of ionic conductivity is shown in the Supporting Information (SI). Since the NE approximations assume no correlations between particles of different species or particles of the same species in the electrolyte, they are intended for dilute or ideal systems. The NE approximation of the partial ionic conductivity of component *i* is
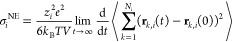
4in which *k*_B_ is the Boltzmann constant, *V* the system volume, and *e* is the elementary charge. The total ionic conductivity is the sum of all the partial conductivity contributions. In a binary electrolyte, the total ionic conductivity based on the NE approximation is
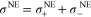
5in which σ_+_^NE^ and σ_–_^NE^ denote the partial conductivity contributions from cations and anions, respectively. The NE approximation of the ion transport number for species *i* is
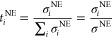
6where we sum over all species in the denominator to obtain the total ionic conductivity, σ^NE^.

In an electrolyte, multiple species are present and attractive, and repulsive interactions will influence the transport of each species. Concentrations deviating from the dilute limit will invalidate some of the assumptions used in the NE approximations. One way to characterize correlations and consider transport properties at higher concentrations is to compute the Onsager coefficients:^8^
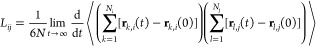
7in which *i* and *j* are components, and *N*_*i*_ and *N*_*j*_ are the numbers of particles of components *i* and *j*, respectively. *N* is the total number of particles in the system. Note that *i* and *j* might denote the same component or different components, and *L*_*ij*_ = *L*_*ji*_. *L*_*ij*_ describes the transport of component *i* in a chemical potential gradient of component *j*. When *i* and *j* are different components, *L*_*ij*_ describes the correlations between the different components. When *i* and *j* are the same component, *L*_*ii*_ includes the self-diffusion contribution but also describes how the motion of other particles of the same component influences the transport of component *i*. A system with *n* components can be described by *n*(*n* – 1)/2 independent Onsager coefficients according to Onsager’s reciprocal relations.^25^ To characterize the transport properties of concentrated electrolytes, the Onsager theoretical framework is more appropriate because it takes into account the importance of coupling and deviations from infinite dilution. Onsager transport theory relates the driving forces acting on the electrolyte species to the flux of the species, with the Onsager coefficient acting as the proportionality constant. The derivation of the Onsager ionic conductivity is shown in the SI.

When we take ionic correlations into account, the partial ionic conductivity contribution from the correlation of species *i* and *j* is
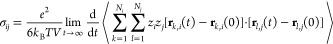
8and the total ionic conductivity is^9,26^
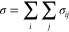
9in which we sum over all ionic pairs in the system. In a binary electrolyte, the total ionic conductivity is
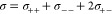
10in which σ_++_, σ_––_, and σ_+–_ denote the partial ionic conductivity contributions from cation–cation correlations, anion–anion correlations, and cation–anion correlations, respectively. In the dilute limit, there are no correlations, and σ_+–_ approaches zero, and σ_++_ and σ_––_ approach σ_+_^NE^ and σ_–_^NE^, respectively. We then obtain the NE approximation of the ionic conductivity, σ^NE^, in eq 5.

The transport number of an ionic species *i* is
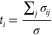
11where ∑_*j*_σ_*ij*_ is the sum of all the partial conductivity contributions of species *i* computed with eq 8. The cation transport number in a binary electrolyte is then:
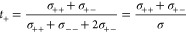
12

## Method

We conducted molecular dynamics simulations with the LAMMPS^27^ software on a nonpolarizable model system composed of SPC/E^13^ water with solvated NaCl^14^ and a polarizable water model SWM4-NDP^15^ with Na^+^ and Cl^–^ ions.^16^ To sample displacement of charged species, we have implemented the *order-n* algorithm of Dubbeldam et al.^28^ as a fix in LAMMPS. In the SPC/E model, the water molecule is rigid with point charges at the atomic positions. The bond length and angle of the water molecule were fixed with the SHAKE algorithm.^29^ The interactions of Na^+^ and Cl^–^ ions were described using the parameters by Weerasinghe and Smith,^14^ which was specifically developed to reproduce the properties of NaCl in water. Long-range Coulombic interactions were treated using standard Ewald summation with relative error in forces of 1 × 10^–5^. Global cutoffs for the Lennard-Jones and Coulombic forces were set to 8 and 12 Å, respectively, and a Lennard-Jones tail correction to the energy and pressure was added.^24^ Geometric mixing rules were used to determine the Lennard-Jones interactions between unlike atoms, except for a special scaled geometric mean of the Lennard-Jones energy parameter for water oxygen and Na^+^. Periodic boundary conditions were applied in all directions. We varied salt concentration and system size to evaluate finite-size effects. Four different salt concentrations were studied, 0.5, 1.0, 2.5, 4.0 mol L^–1^, and system sizes of 800, 3000, 10000, 20000 water molecules were investigated. Packmol^30^ and fftool^31^ were used to prepare initial configurations of pure water. Na^+^ and Cl^–^ ions were placed randomly inside the box with a subsequent energy minimization employing the conjugate gradient algorithm to avoid initial overlap of particles.

Charge transport properties of the nonpolarizable model systems were calculated using equilibrium and nonequilibrium simulations. In the equilibrium simulations, the systems were first equilibrated in the isobaric–isothermal (NPT) ensemble at a temperature of 293 K and pressure of 1 atm for 3 ns with a time step of 1 fs. The Nosé–Hoover thermostat and barostat were used to control the temperature and pressure in the NPT ensemble.^32−34^ The box volume was scaled according to the average volume during the equilibration to obtain correct density in the canonical (NVT) ensemble. After equilibration for 2 ns in the NVT ensemble, transport properties were sampled at a temperature of 293 K during production runs of 1 ns with a time step of 2 fs. The temperature was controlled with the Nosé–Hoover thermostat utilizing a time constant resulting in characteristic thermal fluctuations of 100 timesteps. We used the OCTP module for LAMMPS to compute self-diffusivities and Onsager coefficients of the solutions.^35^ Ionic conductivity and transport numbers were calculated with the Nernst–Einstein and Onsager frameworks, using eqs 4, 6, 9, and 11. The Nernst–Einstein values were corrected for finite-size effects using the Yeh-Hummer correction for self-diffusion coefficients.^36−38^ We made five replicas of each system. The replicas were prepared in the NPT ensemble by heating equilibrated systems from 293 K to 400 K during 10 ps, mixing for 20 ps at 400 K, cooling back to 293 K during 10 ps, and finally mixing at 293 K for 200 ps before saving the final configuration as a replica system. The velocities of all particles were reset before each replica run.

A rigid polarizable water model, SWM4-NDP,^15^ together with polarizable Na^+^ and Cl^–^ ions,^16^ was also investigated to compare with the nonpolarizable model. The SWM4-NDP water model and associated ionic model utilize Drude particles to describe atomic polarizability. A negatively charged Drude particle is attached to the positively charged core particle by a harmonic spring. The polarizability of the atom or ion is adjusted by changing the charge of the Drude (and core) particle.^15^ The original Na^+^ and Cl^–^ ionic model^16^ was parametrized against single ion properties, i.e., the hydration free energy at infinite dilution. Consequently, it did not describe the properties of concentrated solutions accurately. Particularly, the Na^+^ and Cl^–^ interactions were too strong, favoring the formation of ionic clusters.^39^ A later study sought to remedy this by optimizing the ionic parameters.^39^ Two methods for optimizing the parameters are presented in ref (39): by adjusting the *R*_min_^Na^+^Cl^–^^ distance parameter in the Na^+^-Cl^–^ Lennard-Jones potential or by introducing Thole damping of the Na^+^-Cl^–^ interaction. We have chosen to use the adjusted *R*_min_^Na^+^Cl^–^^ value in this work. Note that the Lennard-Jones distance parameter σ as used in LAMMPS is equal to *R*_min_/2^(1/6)^. The Lennard-Jones ionic parameters are in Table 1.

**Table 1:** Lennard-Jones Parameters for the Polarizable Ions with the SWM4-NDP Water Model As Used in LAMMPS

ion(s)	ϵ (kcal/mol)	σ (Å)
Na^+^	0.0315100	2.6044177
Cl^–^	0.0719737	4.4208424
Na^+^-Cl^–^	0.0476224	3.6437758

The Langevin thermostat was used to control the temperature in the polarizable simulations. The center of mass of the Drude-core particles were set to 293 K and the Drude particles to 1 K. To minimize the effect on the particle dynamics, weak Langevin damping coefficients of 10 and 5 ps were used for the motions of the Drude-core center-of-mass and Drude particle relative to the core, respectively.^40^ The Lennard-Jones and Coulombic forces were cut off at 12 Å and long-range Coulombic forces were computed by the particle–particle particle-mesh solver^41^ with relative error in forces of 1 × 10^–6^. A long-range Lennard-Jones tail correction was added to the energy and pressure. The water molecules were held rigid by a special fix in LAMMPS.^42^ Lorentz–Berthelot mixing rules were applied for the interaction between the ions and the water oxygen. The bond between the Cl^–^ core and Drude particle included an anharmonic restoring force for bond lengths above 0.2 Å, as described in ref (16), to avoid the polarization catastrophe often encountered in highly polarizable ions. Initial configurations were prepared similarly to the nonpolarizable model, but without energy minimization after adding the ions. A time step of 0.5 fs was used in all the simulations with the polarizable model. Equilibration was performed in the isobaric–isothermal (NPT) ensemble at 293 K and 1 atm pressure. The Nosé–Hoover barostat controlled the pressure with a relaxation time of 500 time steps. Equilibration was performed for at least 500 ps, during which the box volume was sampled, and at the end adjusted to obtain correct density during sampling with constant volume. It was necessary to reset the linear momentum of the box during the equilibration to avoid the flying ice cube effect, but not during the constant volume simulations.^42^ Sampling of charge transport properties was performed similarly to the nonpolarizable model in the NVT ensemble during simulations of at least 1 ns. Two polarizable systems were studied, with salt concentrations 1.0 and 2.45 mol L^–1^, composed of 9000 water molecules and 167 and 417 NaCl, respectively. We made three replicas of both systems from different initial configurations.

In the nonequilibrium MD simulations (only nonpolarizable model), the systems were initially equilibrated in the NPT ensemble as described above. An external uniform electric field was applied in the *x*-direction after switching to the NVT ensemble. The electric field is invoked as a force **F** = *ze***E** that is applied to each particle in the box, where **E** is the electric field vector. The ions will begin to drift under the influence of the electric field. To avoid influencing the ionic fluxes caused by the electric field, the thermostat was applied to the two other directions than the applied field direction, i.e., the *y*- and *z*-directions, as done in previous studies.^43−45^ The Nosé–Hoover thermostat was employed with a similar time constant as in the equilibrium simulations. After equilibration for 3 ns in the NVT ensemble, data was sampled over 15 ns with a time step of 2 fs to determine ionic conductivity and transport numbers. We applied the method explained by Shen and Hall in section 2 of the SI in ref (46) to compute the ionic conductivity and transport numbers in the nonequilibrium MD simulations. Here, the drift velocities of the ionic species are determined from the so-called field MSDs. The field MSD is the MSD due to the electric field. This is calculated by subtracting the MSD in a simulation without applied field from the MSD in a simulation with applied field. Alternatively, one can subtract the average MSD in the directions perpendicular to the field direction. The ionic drift velocity, *v*_d_, is determined by fitting the field related MSD to a quadratic expression *a* + *bt*^2^, where *v*_d_ = √*b*, as the field MSD has a slope of 2 in a log–log plot at long times.^46^ The ionic mobility is
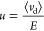
13where *E* is electric field strength. The total ionic conductivity is
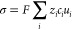
14where *c*_*i*_ is the molar concentration of species *i*.

Several pitfalls of doing nonequilibrium MD simulations are discussed in the literature.^7,47−49^ Notably, the transport properties might depend on the magnitude of the applied field due to nonlinearities. An example of such nonlinear behavior is the evolution of high-speed lanes for ionic species.^47^ Ions then follow in the wake of other ions with similar charge where they experience less friction than outside the lane, and consequently the diffusivities become too large.^7^ To establish if this influenced results to a large degree, we studied the effect of the electric field strength on the transport properties by varying the field strength from 0.01 to 0.05 V Å^–1^ in steps of 0.01 V Å^–1^. The response approaching the zero field limit was examined by evaluating field strengths of 0.005, 0.003, and 0.001 V Å^–1^. Five replicas of the system with 3000 SPC/E water molecules and 140 NaCl were studied with nonequilibrium simulations at each field strength, using the same replicas as those prepared for the equilibrium MD simulations. We also performed nonequilibrium simulations of the systems with 10000 SPC/E water molecules and salt concentrations of 0.5, 1.0, 2.5, and 4.0 mol L^–1^ using an electric field strength of 0.03 V Å^–1^ to evaluate the effect of salt concentration. Three replicas were examined for each salt concentration.

All reported values and uncertainties were estimated by calculating the mean and standard deviations of the quantities obtained from the replicas. The standard deviations of the computed values in the replicas are denoted as error bars in the plots.

## Results and Discussion

### Equilibrium Molecular Dynamics

**Figure 1:**
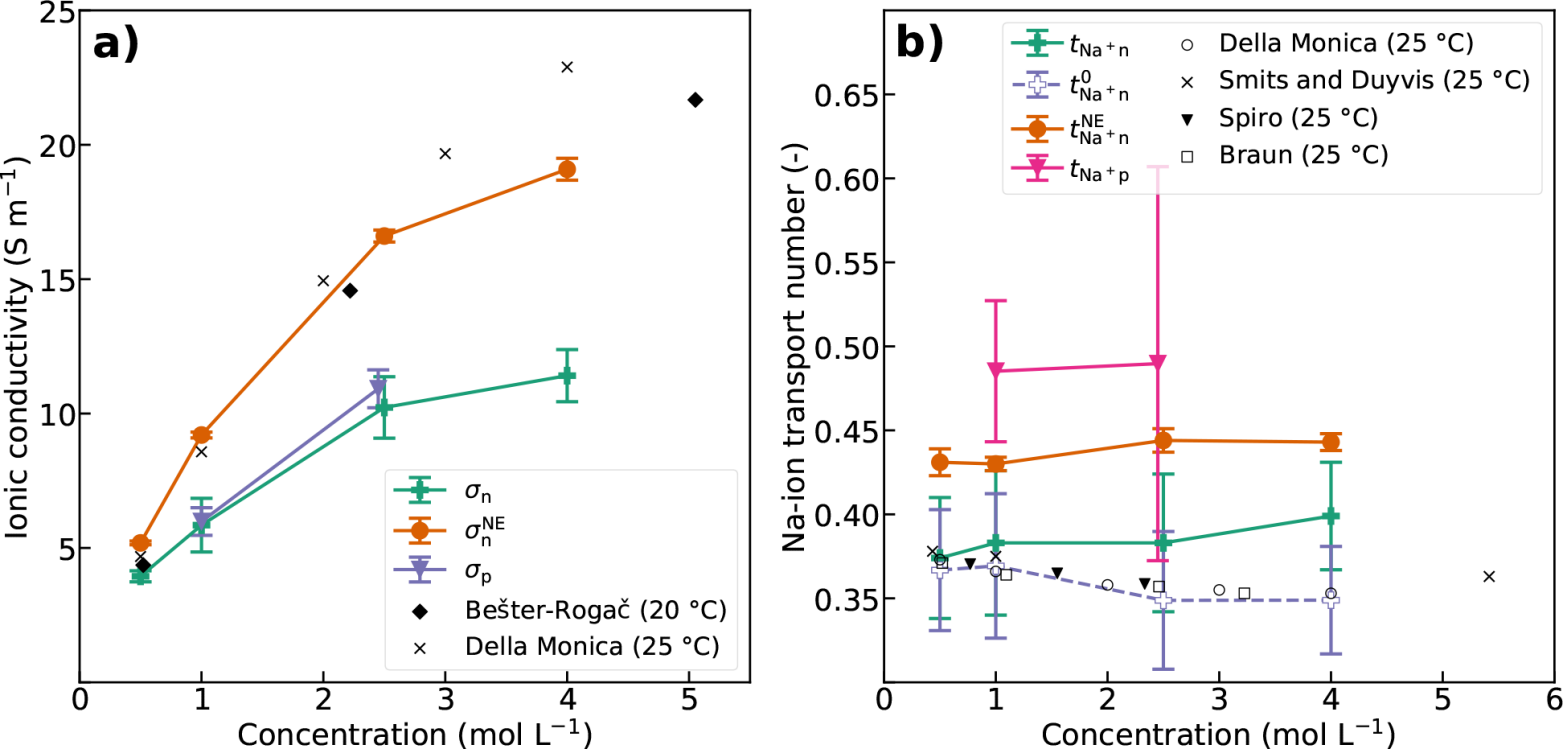
(a) Ionic conductivity and (b) Na-ion transport number as a function of salt concentration from equilibrium MD simulations on the systems with 10000 SPC/E and 9000 SWM4-NDP water molecules compared to experimental data. The results from nonpolarizable (SPC/E water) and polarizable (SWM4-NDP water) simulations are denoted with subscripts n and p, respectively. The computed ionic conductivities are compared to experimental data by Bešter-Rogač et al.^51^ and Della Monica et al.^10^ The computed transport numbers in the barycentric and solvent velocity reference frames are displayed, the latter denoted with superscript 0. The computed Na-ion transport numbers are compared to experimental data by Della Monica et al.,^10^ Smits and Duyvis,^52^ Spiro,^11^ and Braun.^12^

The deviation between the ionic conductivity of the SPC/E model with NaCl computed with Onsager’s theory and experimental data clearly increases with increasing salt concentration. This is not surprising, considering that SPC/E with NaCl is a nonpolarizable force field. It is well-known that nonpolarizable models significantly overestimate ion–ion correlations.^50^ This is shown in Figure 1, which show the ionic conductivities and Na-ion transport numbers computed with the Nernst–Einstein approximation (eqs 4 and 6) and Onsager’s theory (eqs 8, 9, and 11) as a function of salt concentration. The ionic motion and resulting ionic conductivity of the model is lower compared to experimental values, particularly at higher salt concentrations as seen in Figure 1a. A common way of reducing the ion–ion correlations in a nonpolarizable model to obtain more correct transport properties is to scale the ionic charges by a factor of 0.7 to 0.8.^50,53^ We scaled the ionic charges by a factor of 0.8 in the system with 10000 SPC/E water molecules and 465 NaCl (2.5 mol L^–1^) to study the effect on the charge transport properties. There were no substantial differences compared to the systems without charge scaling and the results are shown in the SI. Interestingly, the Nernst–Einstein approximation corresponds better with experimental data. However, this does not mean that the Nernst–Einstein approximation necessarily offers a better description of the ionic conductivity of the SPC/E water + NaCl model. We expect that the model underestimates the ionic conductivity at higher salt concentrations. At lower salt concentrations, both the Onsager and Nernst–Einstein methods are in better agreement with experimental data, as expected.

The polarizable model of SWM4-NDP water and Na^+^ and Cl^–^ ions displays very similar ionic conductivity to the nonpolarizable model. This is surprising as we expect improved description of ion–ion interactions in the polarizable model would result in better agreement with experimental results. As mentioned earlier, the ionic parameters were initially parametrized against single ion properties and were not able to describe concentrated solutions correctly. Later, the interactions between Na^+^ and Cl^–^ in SWM4-NDP water were adjusted to reproduce the osmotic pressure in concentrated solutions.^39^ Obviously, this does not guarantee that transport properties, such as the ionic conductivity, and ionic correlations are perfectly described.

The computed ion transport numbers in the nonpolarizable model in Figure 1b are in good agreement with the experimental data at all salt concentrations studied. We observe a similar trend here as with the ionic conductivity, the discrepancy between the computed values and experimental values increase with increasing salt concentration. The transport numbers obtained by the Onsager equation agrees better with experimental data than the Nernst–Einstein approximation. Notably, the error bars for the Onsager ionic conductivity and transport numbers are significantly larger than the Nernst–Einstein approximations. The cause for this is clear when comparing the corresponding equations for ionic conductivity, eqs 9 and 4. In the equation for NE ionic conductivity, eq 4, we average over all ions of each kind, but the Onsager ionic conductivity, eq 9, is more susceptible to statistical noise and has less statistical data. The calculations of ion transport numbers are affected in the same way.

The Na-ion and Cl-ion transport numbers are almost equal, about 0.5, at both salt concentrations 1.0, 2.45 mol L^–1^ in the polarizable model. The polarizable model produces less correct transport numbers than the nonpolarizable model, compared to experimental data. The Na^+^ and Cl^–^ ions apparently move almost equally fast in the polarizable model. We will not try to explain the reason for this behavior. Since the nonpolarizable model gives the most accurate results, we choose to focus on this model for the remainder of the article.

Determining ion transport numbers depends on the frame of reference, which will depend on the experimental or simulation method. The experimental values we compare our results with are obtained with different methods. For example, Della Monica et al.^10^ and Smits and Duyvis^52^ used the Hittorf and emf methods, respectively, which both employ the solvent velocity reference frame;^52,54^ i.e., the transport numbers are determined with respect to the solvent. For MD simulations, the center of mass of all particles in the simulation box is the frame of reference, i.e., the barycentric reference frame. If the center of gravity of the electrolyte does not move relative to the solvent, the solvent velocity and barycentric reference frames are equivalent. This is likely the situation in dilute electrolytes, but it might not be the case in highly concentrated electrolytes where the ions make up a large part of the total mass. Either way, it is important to understand the significance of the reference frame when analyzing and comparing transport numbers. Barycentric transport numbers can be readily transformed to solvent velocity transport numbers.^55^ We transformed the transport numbers from the nonpolarizable model computed with Onsager coefficients to the solvent velocity reference frame and obtained very good agreement with the experimental data which are mostly measured in this reference frame. The results are shown in Figure 1b. For a more extensive discussion on reference frames and how to transform between different reference frames, we refer the reader to refs (25) and (55).

**Figure 2:**
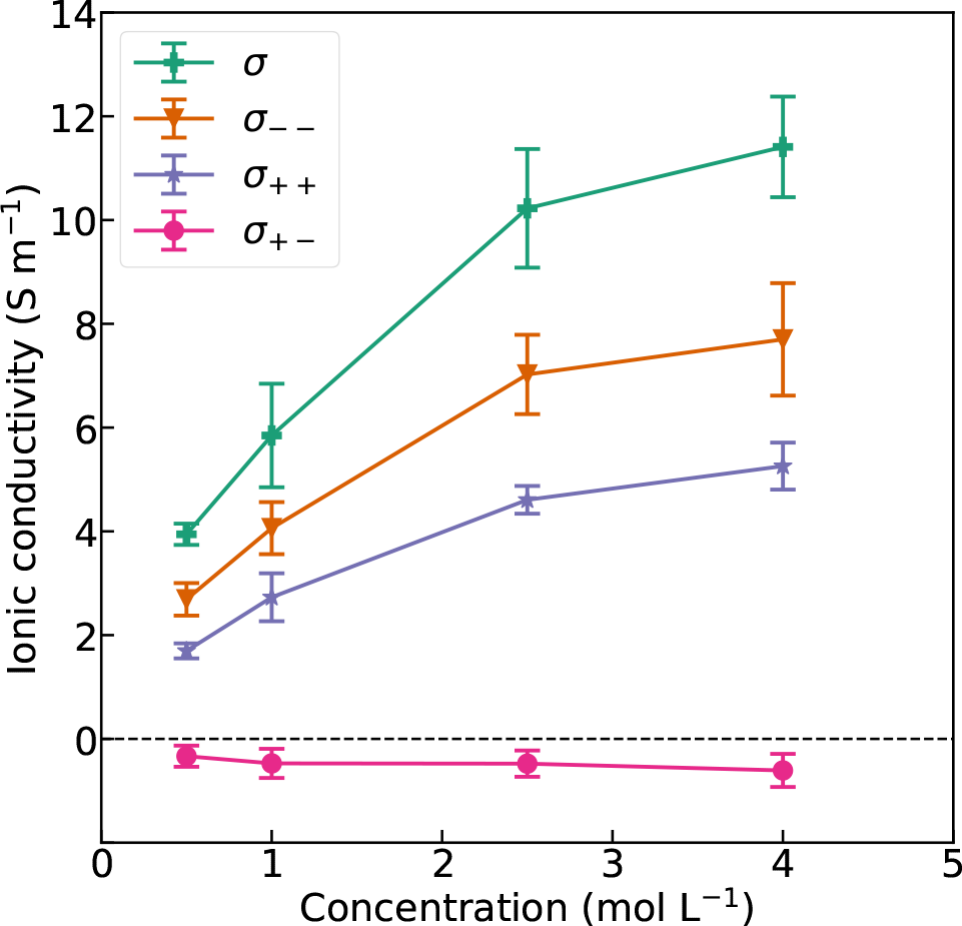
Ionic conductivity contributions as a function of salt concentration in the nonpolarizable model.

Figure 2 shows the decomposed and total ionic conductivities as a function of salt concentration. A larger σ_––_ than σ_++_ means that the Cl^–^ ions will move further in the electrolyte than the Na^+^ ions, and the Na-ion transport number is therefore below 0.5. We can explain this by considering ionic radii. Na^+^ is a smaller ion than Cl^–^ and will have a higher charge density. In water the ions will be surrounded by a solvation shell of coordinating water molecules. Ions with higher charge density will be more strongly coordinated by more water molecules resulting in a larger effective radii which will reduce the ionic mobility compared to ions with lower charge density.^56^ This is reflected in the respective radial distribution functions (RDF) of Na/Cl and oxygen from water, which are displayed in Figures S1 and S2, respectively. The RDF of Na and O displays a higher peak at a shorter interatomic distance than the RDF of Cl and O, which means that there are more water molecules closer to Na than Cl. The cross-correlation σ_+–_ is slightly negative and reduces the total ionic conductivity. The reason is that the oppositely charged Na^+^ and Cl^–^ ions attract each other and will slow each other down when passing. The effect is rather small because of the surrounding water molecules which effectively screen the electrostatic charges due to the high relative permittivity (dielectric constant) of water.^57^ The negative σ_+–_ explains why the Nernst–Einstein approximations of the cation transport number are larger than the corresponding Onsager values, as shown in Figure 1b. Since σ_++_ is smaller than σ_––_, a negative σ_+–_ will cause a reduction of the cation transport number compared to the Nernst–Einstein approximation, according to eq 12.

**Figure 3:**
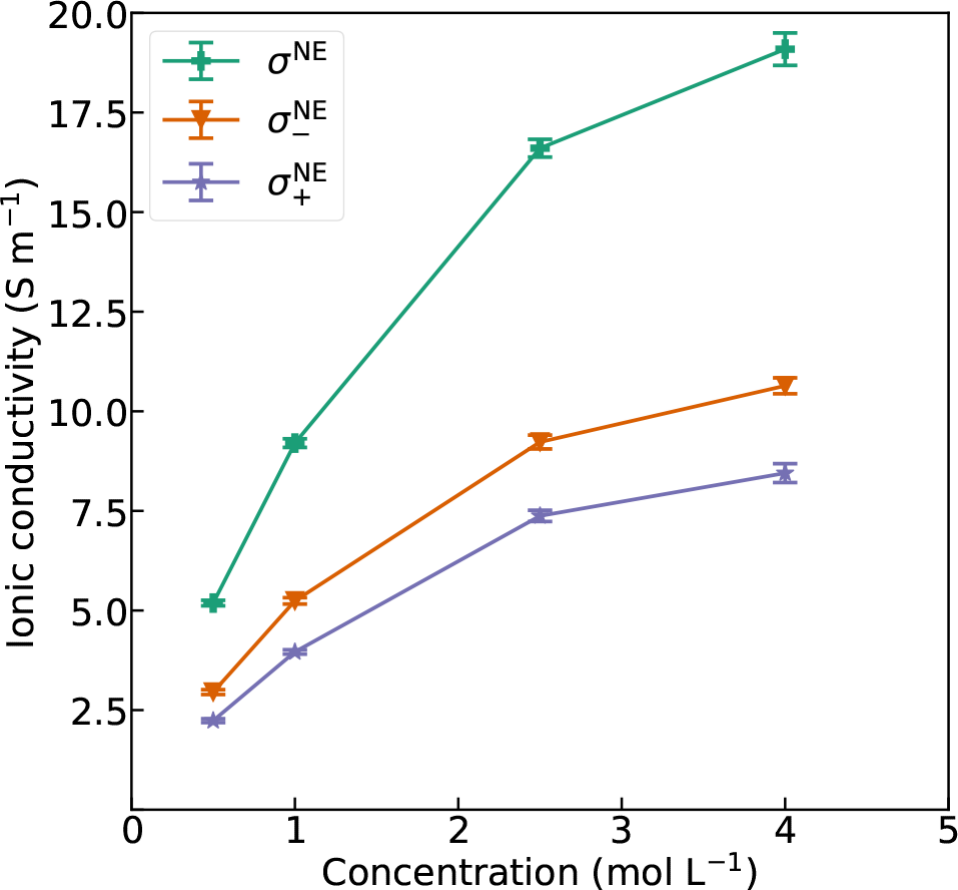
NE ionic conductivity contributions as a function of salt concentration in the nonpolarizable model.

The Nernst–Einstein approximations of decomposed and total ionic conductivities as a function of salt concentration are displayed in Figure 3. These data are based on the self-diffusion coefficients and describe the ionic conductivity assuming ideal conditions, but they do not provide any information about cross-correlations between the ions.

The finite-size effects on ionic conductivity and transport numbers were small and are shown in Figures S3 and S4. Example log–log plots of the NE conductivity MSDs; MSD_+_^NE^, and MSD_–_^NE^, and the conductivity MSDs; MSD_++_, MSD_––_, and MSD_+–_ obtained from equilibrium MD simulations are shown in Figures S5 and S6, respectively.

### Nonequilibrium Molecular Dynamics

**Figure 4:**
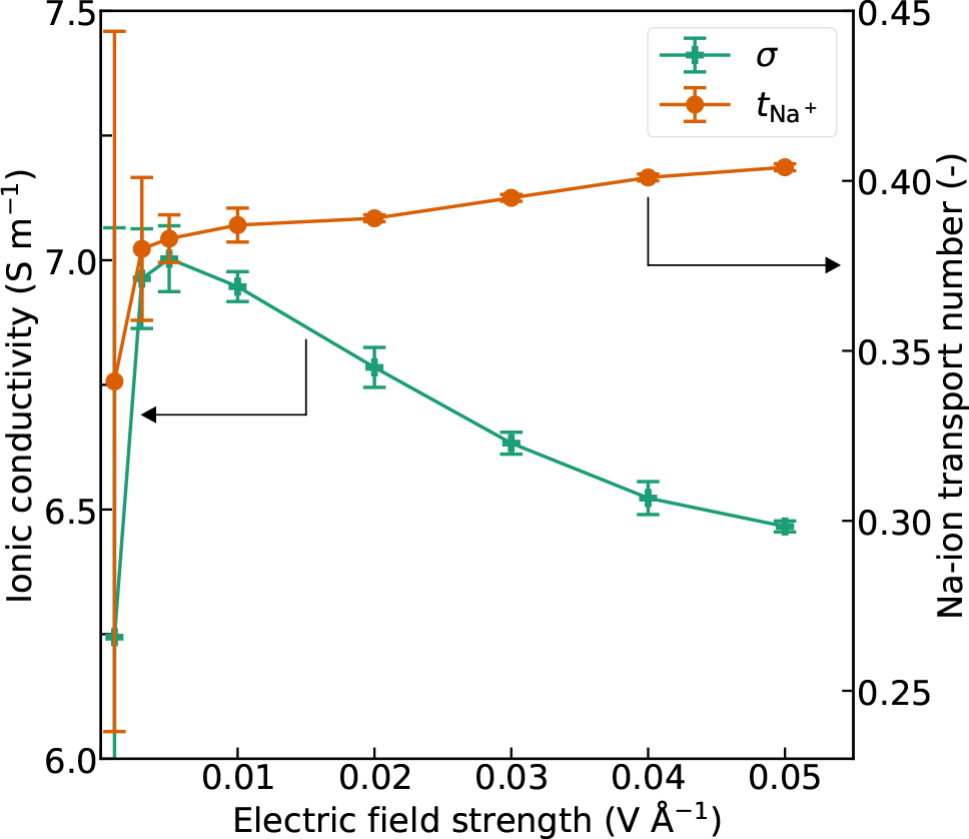
Ionic conductivity and Na-ion transport number as a function of electric field strength in the system with 3000 SPC/E water molecules and 140 NaCl.

Nonequilibrium MD simulations to study the effect of electric field strength were conducted on the system with 3000 water molecules and 140 NaCl molecules, corresponding to a salt concentration of 2.5 mol L^–1^. We confirmed that the simulations were done in the linear response regime by plotting the ion drift velocity against electric field strength. The plot verifies a linear relation and is presented in Figure S7. Ionic conductivity and transport numbers are strictly defined when the concentration is uniform. This might not be the case when an external electric field is applied to the simulation box. However, after a steady-state ionic drift is established, any concentration gradients will be small, and thus allow the use of this method with small electric fields. Figure 4 shows the computed ionic conductivity and Na-ion transport number as a function of the electric field strength. The ionic conductivity computed with the nonequilibrium method is lower than the equilibrium results. Additionally, the ionic conductivity decreases slightly with increasing electric field strength for field strenghts above 0.01 V Å^–1^. The strength of the applied electric field is considerable, roughly 3 orders of magnitude higher than in for example a typical battery electrolyte. The water molecule, due to its dipole moment, will likely be strongly polarized and aligned in the field, which will limit its self-diffusivity and rotational motion as shown in several studies.^58−62^ This could in turn impede the mobility of the ions and reduce the ionic conductivity. The effect might be present also with other small polar solvent molecules in a simulated static electric field. The Na-ion transport number increases with increasing field strength but appears to approach the equilibrium value including uncertainty (Figure S4) upon extrapolation of the linear part of the curve to 0 V Å^–1^ field strength. As the Cl^–^ are heavier and move faster than the Na^+^, the linear momentum due to the ionic fluxes will not cancel out. In order to compensate for this, the water molecules will gain a momentum in the same direction as the Na^+^, which will increase with the magnitude of the field. The influence of water molecules moving in the same direction as the Na^+^ could explain why the Na-ion transport number increases with increasing field strength. The ionic conductivity decreases when approaching the zero-field limit. Considerable field strengths are necessary to establish ionic fluxes at the time scales of nonequilibrium simulations.^7,49^ The ionic fluxes will diminish as the slopes of the field MSDs approach 1 when the field becomes too weak, and consequently the ionic conductivity is reduced. The signal-to-noise ratio decreases and the uncertainty increases upon approaching 0 V Å^–1^ field strength. The linear part of the ionic conductivity curve does not approach the equilibrium value upon extrapolation to the zero-field limit which suggests that the field strength needed to establish ionic fluxes is larger than the field strength required to orient the water molecules.

The average temperature of the box, specifically the temperature in the direction of the field, went down with increasing field strength. This effect is displayed in Figure S8. The temperature drop in the field direction was about 11 K at the highest field strength (4 K average temperature drop), which might contribute to the reduced ionic conductivity with increasing field strength.^51^ This is likely an anomaly due to the use of rigid molecules in an applied field. The number of degrees of freedom is reduced from nine to six in the rigid SPC/E water molecule, due to the two frozen bonds and frozen angle. When subjected to a field, we believe the rigidity or reduced number of degrees of freedom of the SPC/E water restricts its rotational motion in the direction of the field, which causes the temperature to decrease. The degrees of freedom removed by the SHAKE algorithm are accounted for in the temperature computation in equilibrium simulations. However, when an external field is applied in the nonequilibrium simulations, it appears the system is further constrained in the field direction which results in a temperature reduction.^63^ With increasing field strength, the effective number of degrees of freedom removed increases causing the temperature to decrease proportionally to the field intensity, as shown in Figure S8. In order to test this hypothesis, we conducted similar simulations but used the flexible three-point SPC/Fw^64^ and four-point TIP4P/2005f^65^ water models instead of rigid SPC/E. The bonds and angles are described using harmonic potentials in SPC/Fw, and Morse and harmonic potentials, respectively, in TIP4P/2005f. We did not observe any temperature drop with increasing field strength using the TIP4P/2005f model and only a very slight temperature reduction with SPC/Fw of about 1 K in the field direction at the highest field strength. This strengthens our hypothesis that the temperature drop was due to the rigidity and reduced degrees of freedom of the SPC/E water molecule which artificially restricts its motion in the electric field direction. The observed temperature drop could be caused by reduced entropy in the field, a phenomenon also observed in other classes of materials, such as ferroics.^66,67^ It is important to note that the temperature effect is different from the effect of limited diffusivity and rotational motion of the water molecules which we believe is the main factor reducing the ionic conductivity.

**Figure 5:**
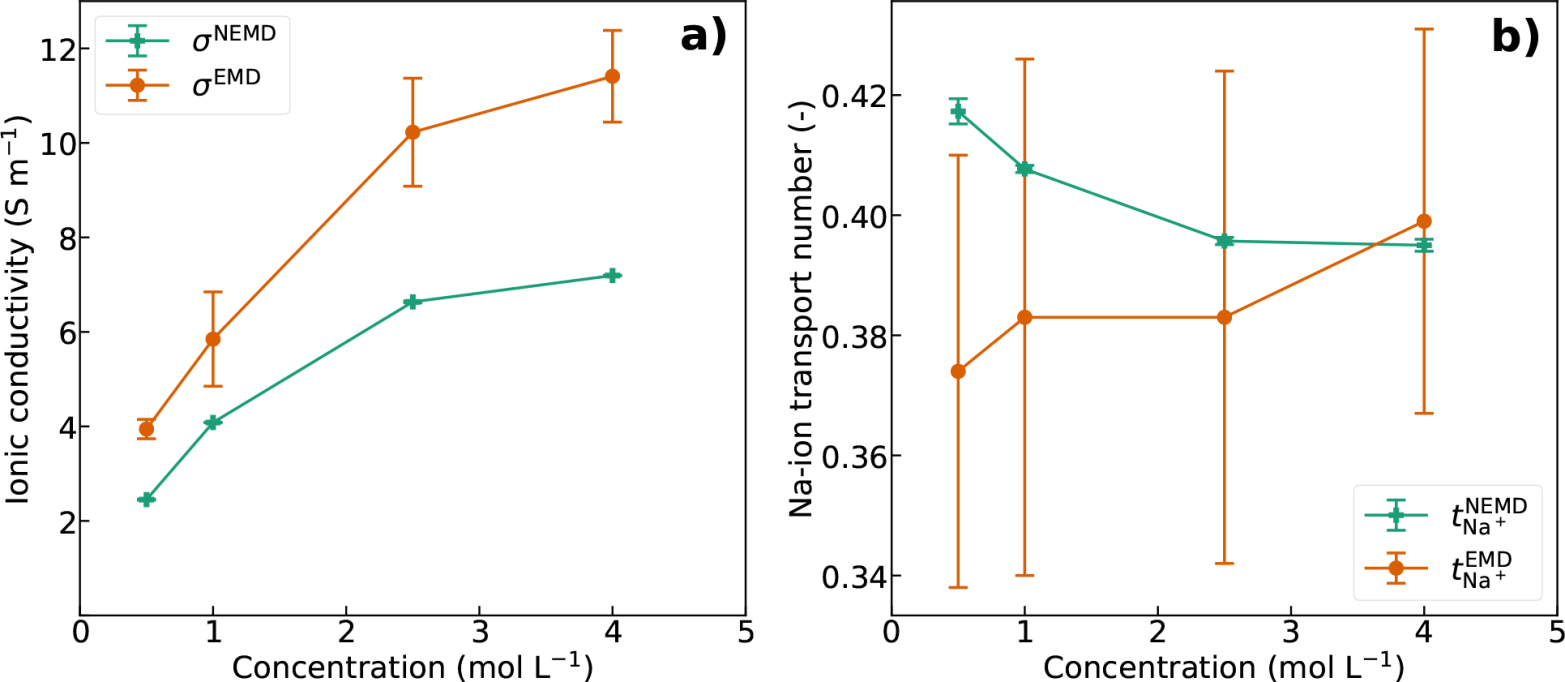
(a) Ionic conductivity and (b) Na-ion transport number as a function of salt concentration in the system with 10000 SPC/E water molecules in nonequilibrium simulations (NEMD) using an electric field strength of 0.03 V Å^–1^. The values are compared to the equilibrium results (EMD) for the same systems.

The ionic conductivity and Na-ion transport numbers as a function of salt concentration for the systems with 10000 water molecules in nonequilibrium simulations are displayed in Figure 5. Again, the ionic conductivity is lower than in the equilibrium simulations, due polarized water molecules aligned to the field that hinder the ionic motion. The relative deviation between the nonequilibrium and equilibrium results does not change much with increasing salt concentration. The nonequilibrium Na-ion transport numbers decrease slightly with increasing salt concentration. As the number of ions increase, the effect of water molecules moving in the same direction as the Na^+^ might be reduced.

It is not possible to obtain data on the main coefficients, σ_++_ and σ_––_, or the ionic cross-correlation, σ_+–_, directly from single field-driven nonequilibrium simulations as we have conducted here. An example log–log plot of MSDs from a nonequilibrium simulation is shown in Figure S9.

## Conclusion

In this work, we have demonstrated that two methods employing equilibrium and nonequilibrium molecular dynamics, respectively, can be used to determine ionic conductivity and transport numbers in a model electrolyte of SPC/E water with NaCl. From both methods we find results that are comparable to experimental data. We have presented the Nernst–Einstein and Onsager frameworks for determining charge transport properties and discuss their advantages and disadvantages. We argue that the Onsager framework can be used to study concentrated electrolytes where ionic correlations are significant. We have shown how the data from these methods can be used to analyze the charge transport properties and relate them to molecular interactions. The importance of the reference frame when determining and comparing transport numbers is emphasized. To compare with the nonpolarizable model of SPC/E and NaCl, we performed equilibrium simulations with a polarizable model of SWM4-NDP water and polarizable Na^+^ and Cl^–^ ions. The polarizable model did not display improved transport properties compared to the nonpolarizable model. Clearly, correctly modeling the charge transport properties in concentrated salt water solutions is challenging. Potential challenges and anomalies related to using nonequilibrium MD simulations to obtain these properties are discussed. Notably, in the SPC/E-NaCl system, the temperature drops with increasing electric field strength due to reduced degrees of freedom in the rigid water molecules. We recommend equilibrium simulations to investigate charge transport properties due to their simplicity relative to nonequilibrium simulations and the possibility of obtaining more information about ionic correlations.

## Data Availability

LAMMPS input-files and initial coordinates for the water/ion-mixtures used in this work can be found at: dx.doi.org/10.5281/zenodo.7691586.

## Supporting Information Available

Derivation of the Nernst−Einstein equation and the equation for Onsager ionic conductivity, as well as additional plots of radial distribution functions and mean squared displacements (PDF)
