# Supplementary material for: Charge Transport in Water–NaCl Electrolytes with Molecular Dynamics Simulations
Source: J Phys Chem B. 2023 Mar 15;127(12):2729–38. doi: 10.1021/acs.jpcb.2c08047 (PMC10068734; doi:10.1021/acs.jpcb.2c08047)
Supplement: Supplementary file 1 — jp2c08047_si_001.pdf [file jp2c08047_si_001.pdf]

Supporting Information: Charge Transport in Water-NaCl Electrolytes with Molecular Dynamics Simulations

Øystein Gullbrekken,† Ingeborg Treu Røe,†,‡ Sverre Magnus Selbach,† and Sondre Kvalvåg Schnell*,†

†*Department of Materials Science and Engineering, Norwegian University of Science and Technology, NTNU, Trondheim NO-7491, Norway*

‡*Current address: SINTEF Energy Research, Trondheim NO-7036, Norway*

E-mail: sondre.k.schnell@ntnu.no

Derivation of the Nernst-Einstein ionic conductivity

The Nernst-Einstein equation for charged particles relates the diffusion coefficient to the mobility[S1]:

$$D_i = \frac{u_i RT}{z_i F}, \tag{1}$$

in which D_i is the diffusion coefficient of species i, R is the gas constant, T is absolute temperature, F is Faraday's constant, z_i is the charge valency of species i, and u_i is the mobility of species i, defined as:

$$u = \frac{\langle v_d \rangle}{E}, \tag{2}$$

where $\langle v_d \rangle$ is the average ionic drift velocity, and E is electric field strength. The partial ionic conductivity of species i in a material is[S2]:

$$\sigma_i = z_i F c_i u_i, \tag{3}$$

in which c_i is the molar concentration of species i. By substituting the expression for u_i in equation (1) into equation (3), we obtain the Nernst-Einstein approximation of the partial ionic conductivity of species i:

$$\sigma_i^{\mathrm{NE}} = \frac{z_i^2 F^2 c_i D_i}{RT}. \tag{4}$$

By substituting the expression for the self-diffusion coefficient into equation (4), we obtain the final expression for the NE approximation of partial ionic conductivity of species i:

$$\sigma_i^{\mathrm{NE}} = \frac{z_i^2 e^2}{6 k_{\mathrm{B}} T V} \lim_{t\to\infty} \frac{\mathrm{d}}{\mathrm{d}t} \left\langle \sum_{k=1}^{N_i} \left(\mathbf{r}_{k,i}(t) - \mathbf{r}_{k,i}(0)\right)^2 \right\rangle, \tag{5}$$

in which k_{B} is the Boltzmann constant, V is volume and e is the elementary charge. Here, we also utilized that $F = eN_A$ and $c_i = N_i/(N_A V)$, where N_A is Avogadro's constant. It is not uncommon to express the NE ionic conductivity in terms of the charge of species i, q_i, which is equal to $z_i e$. The total ionic conductivity is the sum of all the partial conductivity

contributions.

Derivation of the Onsager ionic conductivity

The flux of a species i is a linear combination of the forces acting on it[S3]:

$$J_i = \sum_j L_{ij} X_j, \tag{6}$$

in which X_j are the forces and L_{ij} are the Onsager coefficients. In an electrochemical cell, the force acting on the species is the negative gradient of the electrochemical potential, $\nabla\bar{\mu}_j = \nabla\mu_j + z_j F \nabla\phi$, where μ_j is chemical potential of species j and ϕ is electric potential[S4]. The ionic conductivity is defined in the absence of concentration gradients[S5]. Typically, the ionic conductivity of an electrolyte is measured using electrochemical impedance spectroscopy where the concentration is assumed to be uniform. We also assume no gradients in temperature or pressure. Hence, we assume no chemical potential gradient:

$$\nabla\bar{\mu}_j = \nabla\mu_j + z_j F \nabla\phi = z_j F \nabla\phi. \tag{7}$$

Substituting for the force X_j in equation (6)[S6]:

$$J_i = -\frac{1}{RT} \sum_j L_{ij} \nabla\bar{\mu}_j = -\frac{F}{RT} \sum_j L_{ij} z_j \nabla\phi. \tag{8}$$

The electric current density can be expressed in terms of the flux of ions:

$$j = F \sum_i c_i z_i J_i, \tag{9}$$

in which c_i is the molar concentration of species i and we sum over all species. Substituting for J_i:

$$j = -\frac{F^2}{RT} \sum_i \sum_j c_i z_i z_j L_{ij} \nabla\phi. \tag{10}$$

The ionic conductivity expressed by Ohm's law is[S5]:

$$j = -\sigma \nabla \phi, \tag{11}$$

where we also assume no concentration gradients. By substituting for j into equation (10):

$$\sigma = \frac{F^2}{RT} \sum_i \sum_j c_i z_i z_j L_{ij}, \tag{12}$$

$$\sigma = \frac{N e^2}{k_\mathrm{B} T V} \sum_i \sum_j z_i z_j L_{ij}, \tag{13}$$

and we obtain an expression equivalent to equation (9) in the main text. N is the total number of particles in the system, equal to $\sum_i N_i$.

Effect of charge scaling

The effect of ionic charge scaling on the transport properties was tested on a system with 10 000 SPC/E water molecules and 465 NaCl ($2.5\,\mathrm{mol\,L^{-1}}$) by reducing the ionic charges to ± 0.8 in an equilibrium simulation. The resulting ionic conductivity was $10.96\,\mathrm{S\,m^{-1}}$ and the Na-ion transport number was 0.32. The ionic conductivity was the same as without charge scaling while the Na-ion transport number was a bit lower.

The error bars in the following figures denote the standard deviation of the computed quantities from five replicates.

Table S1: Systems studied with equilibrium molecular dynamics simulations. Concentration effects were evaluated for the system containing 10 000 SPC/E water molecules.

No of water molecules	No of NaCl	Salt concentration ($\mathrm{mol\,L^{-1}}$)	Polarizable
800	10	0.5	No
800	50	2.5	No
3000	28	0.5	No
3000	140	2.5	No
10 000	93	0.5	No
10 000	186	1.0	No
10 000	465	2.5	No
10 000	744	4.0	No
20 000	186	0.5	No
20 000	930	2.5	No
9000	167	1.0	Yes
9000	417	2.45	Yes

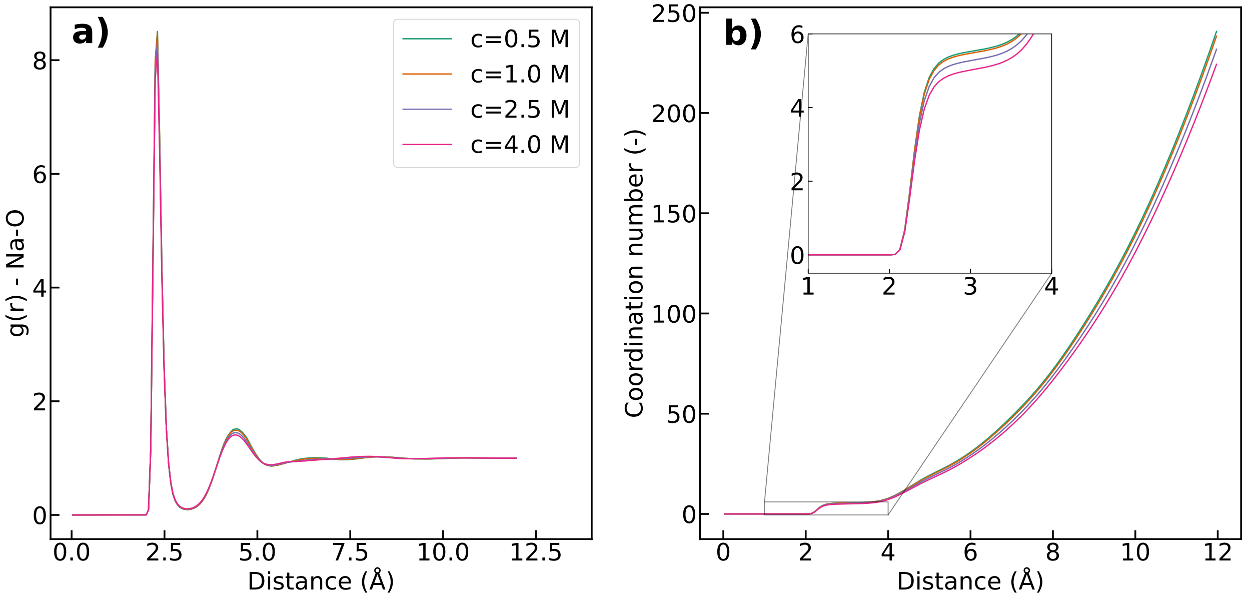

Figure S1: (a) Radial distribution functions and (b) coordination numbers of Na and water oxygen for the different salt concentrations. Data obtained from the systems with 10 000 SPC/E water molecules.

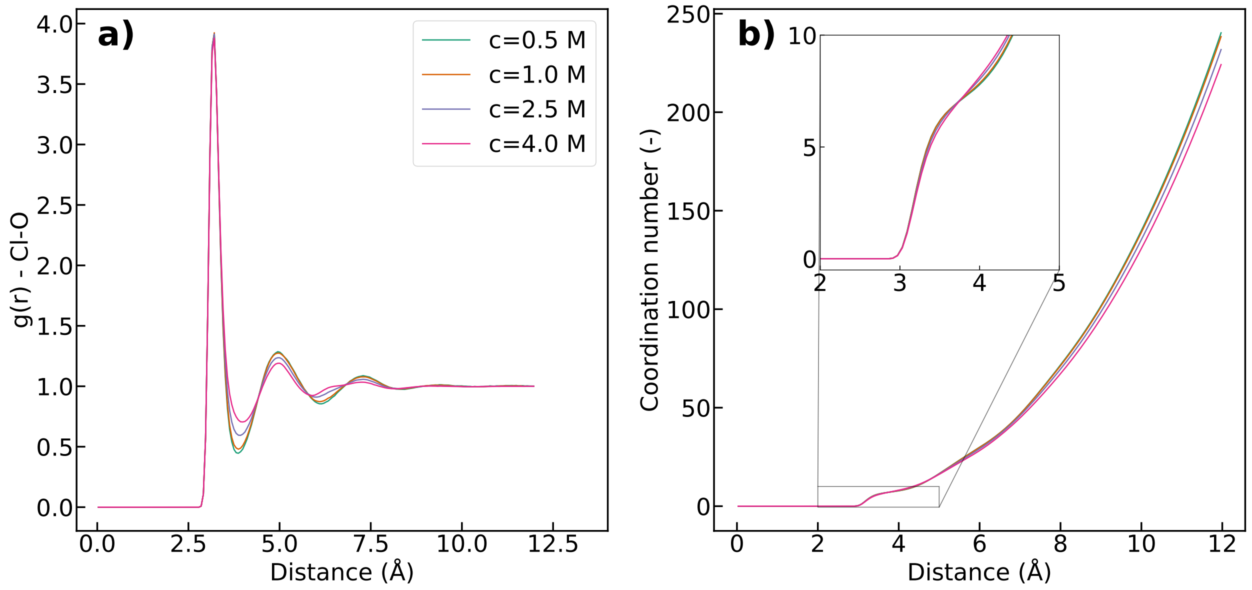

Figure S2: (a) Radial distribution functions and (b) coordination numbers of Cl and water oxygen for the different salt concentrations. Data obtained from the systems with 10 000 SPC/E water molecules.

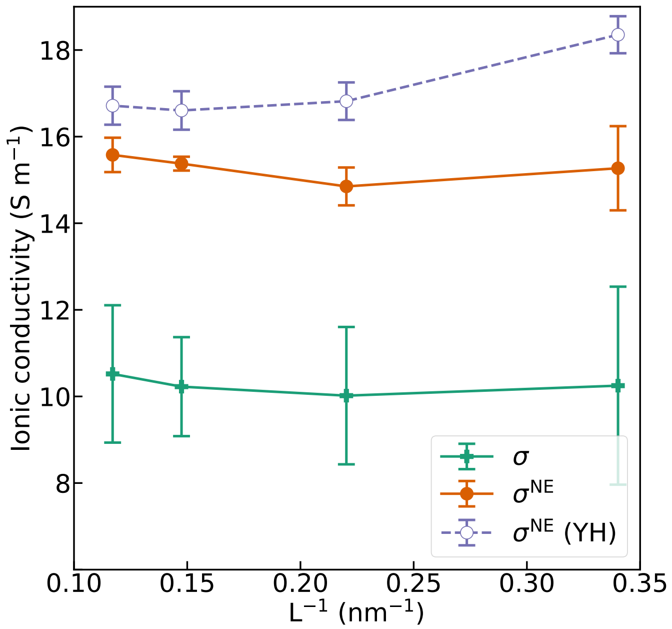

Figure S3: Ionic conductivity plotted against inverse of box length (cubic box). The data points from left to right correspond to the systems with 20 000, 10 000, 3000 and 800 SPC/E water molecules with salt concentration of 2.5 mol L^{-1}. Finite-size corrected values using the Yeh-Hummer (YH) correction are displayed.

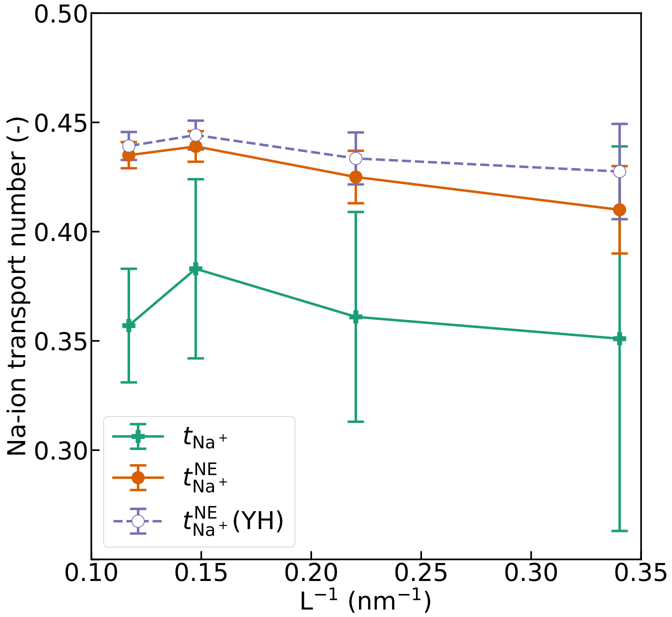

Figure S4: Na-ion transport numbers plotted against inverse of box length (cubic box). The data points from left to right correspond to the systems with 20 000, 10 000, 3000 and 800 SPC/E water molecules with salt concentration of 2.5 mol L^{-1}. Finite-size corrected values using the Yeh-Hummer (YH) correction are displayed.

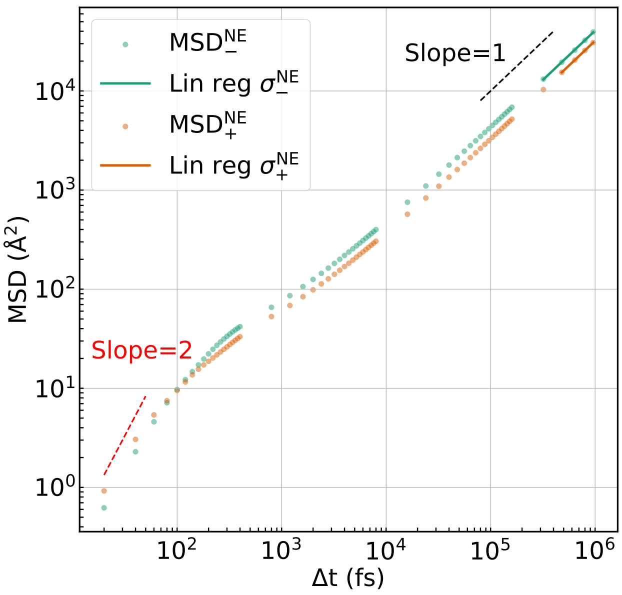

Figure S5: Example log-log plot of output NE conductivity MSD values from the equilibrium simulations. Note that Δt on the x axis denotes time difference, not simulation time. Linear regression to determine the σ^{NE} values is also shown. The data come from the system containing 10 000 SPC/E water molecules and 465 NaCl, corresponding to a concentration of 2.5 mol L^{-1}. Note the ballistic region with slope ∼2 up to about 60 fs.

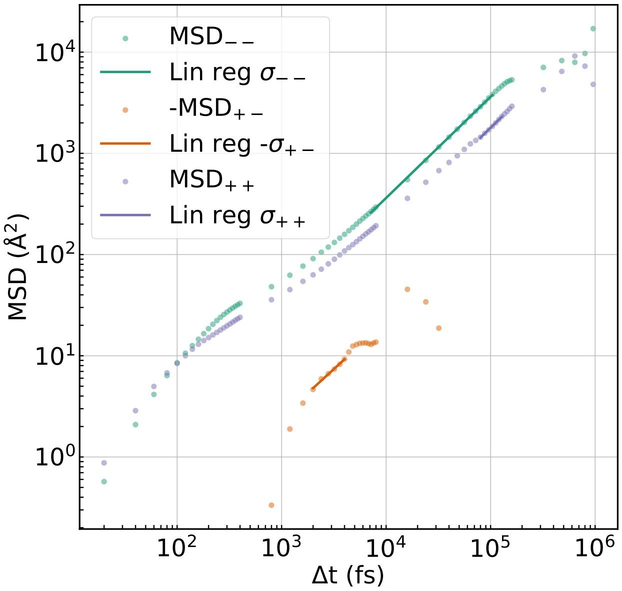

Figure S6: Example log-log plot of output conductivity MSD values from the equilibrium simulations. Linear regression to determine the σ values is also shown. The data come from the system containing 10 000 SPC/E water molecules and 465 NaCl, corresponding to a concentration of $2.5\,\mathrm{mol\,L^{-1}}$.

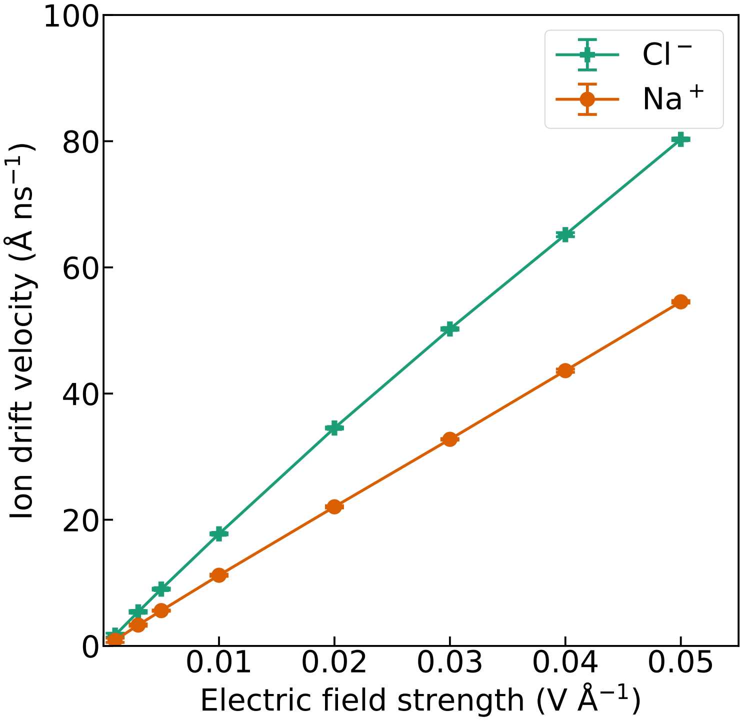

Figure S7: Ion drift velocity as function of electric field strength in the non-equilibrium simulations.

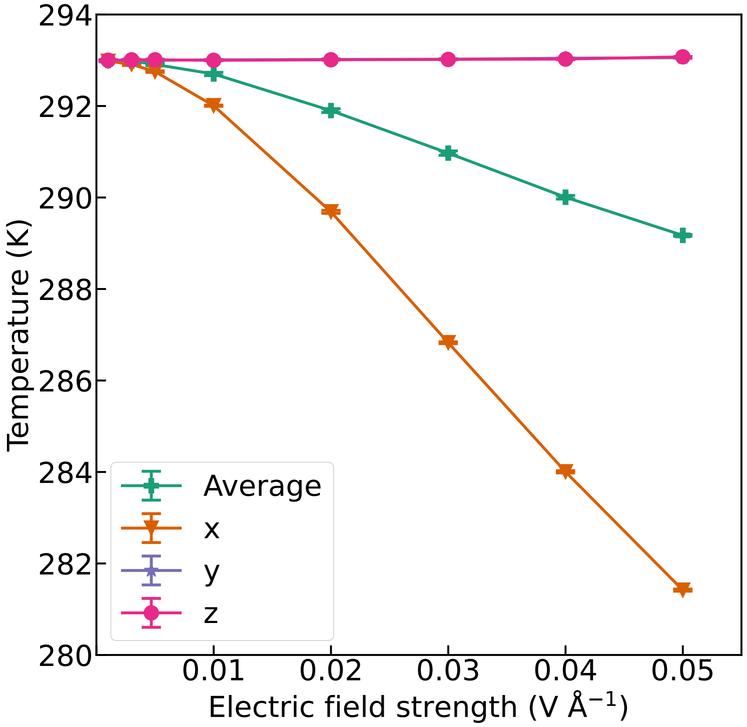

Figure S8: Average temperature and temperature in the three dimensions as function of electric field strength in the non-equilibrium simulations.

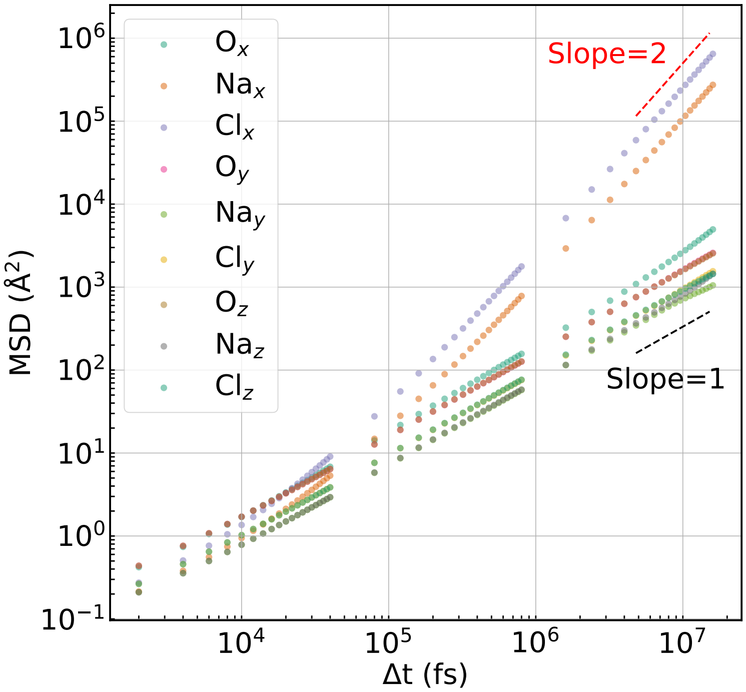

Figure S9: Example log-log plot of MSD values of each component in a non-equilibrium simulation with applied electric field. The data come from one of the replicates of the system containing 3000 SPC/E water molecules and 140 NaCl with an applied electric field of $0.03\,\mathrm{V\,\mathring{A}^{-1}}$. O signifies the oxygen atom in water. MSD values are shown for each dimension, x, y and z, individually. The MSDs of Na^+ and Cl^- in the field direction have slope $\sim$2 at longer times while all the other MSDs have slope $\sim$1.

References

(S1) Frenkel, D.; Smit, B. *Understanding Molecular Simulation (Second Edition)*; Academic Press: San Diego, 2002.

(S2) Rao, C. N. R.; Gopalakrishnan, J. *New Directions in Solid State Chemistry*, 2nd ed.; Cambridge University Press, 1997.

(S3) Kjelstrup, S.; Bedeaux, D. *Non-Equilibrium Thermodynamics of Heterogeneous Systems*; World Scientific: Singapore, 2008.

(S4) Fong, K. D.; Bergstrom, H. K.; McCloskey, B. D.; Mandadapu, K. K. Transport phenomena in electrolyte solutions: Nonequilibrium thermodynamics and statistical mechanics. *AIChE Journal* **2020**, *66*, e17091.

(S5) Newman, J. *Electrochemical Systems*, 2nd ed.; Prentice-Hall, 1991.

(S6) Krishna, R.; Wesselingh, J. The Maxwell-Stefan approach to mass transfer. *Chemical Engineering Science* **1997**, *52*, 861–911.
